# Supplementary figures and images for: Enhancing Oral Delivery of Biologics: A Non-Competitive and Cross-Reactive Anti-Leptin Receptor Nanofitin Demonstrates a Gut-Crossing Capacity in an Ex Vivo Porcine Intestinal Model
Source: Pharmaceutics. 2024 Jan 16;16(1):116. doi: 10.3390/pharmaceutics16010116 (PMC10820293; doi:10.3390/pharmaceutics16010116)

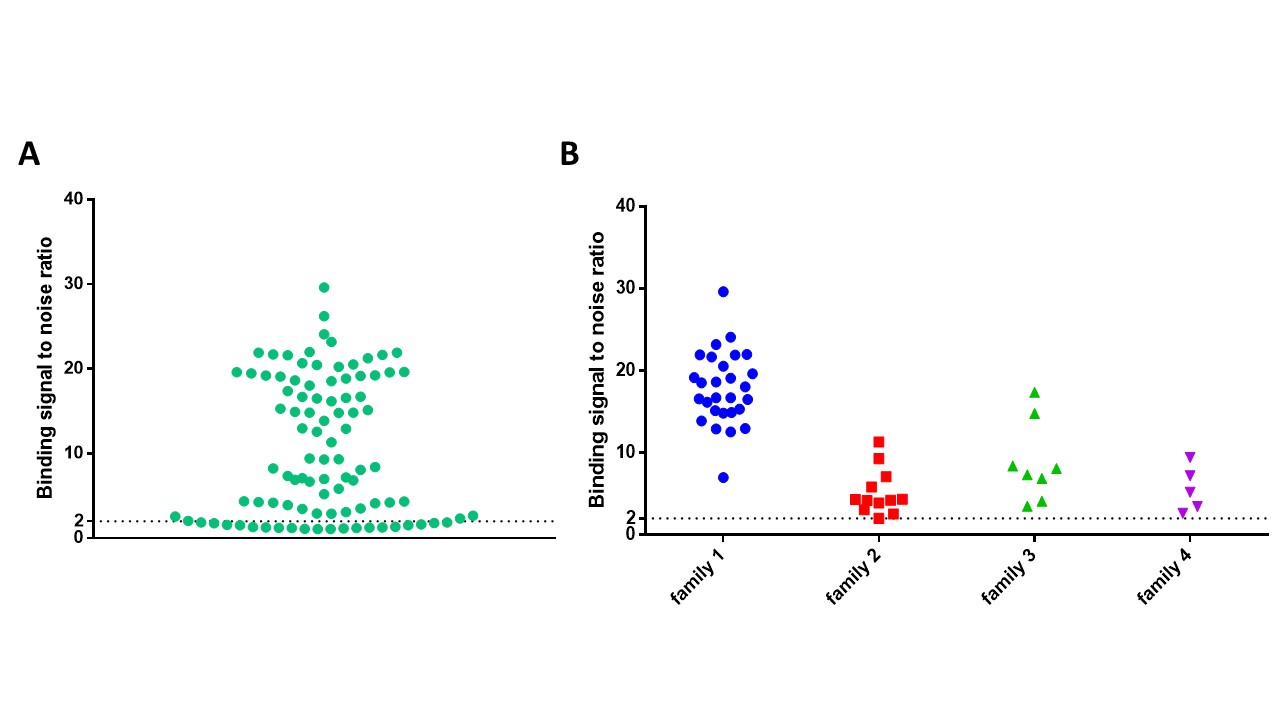

Supplement: Supplementary file 1 [file pharmaceutics-16-00116-s001.zip › Figure S1.jpg]

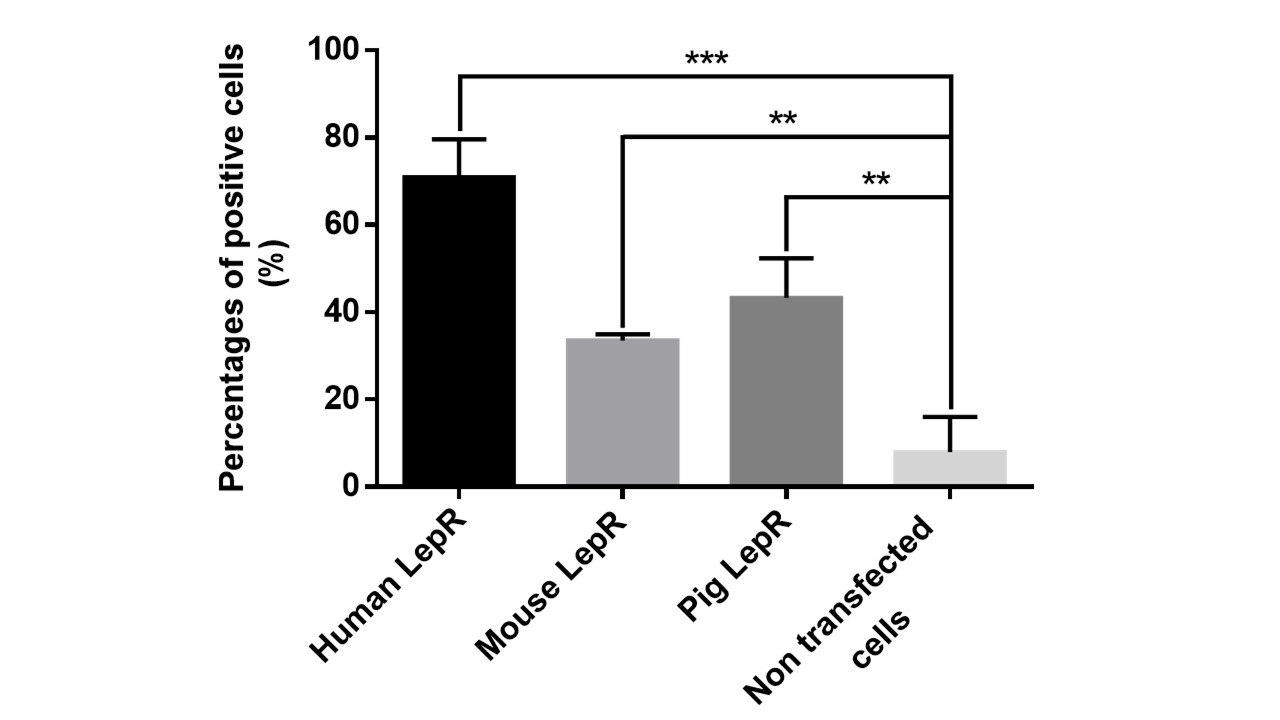

Supplement: Supplementary file 1 [file pharmaceutics-16-00116-s001.zip › Figure S2.jpg]

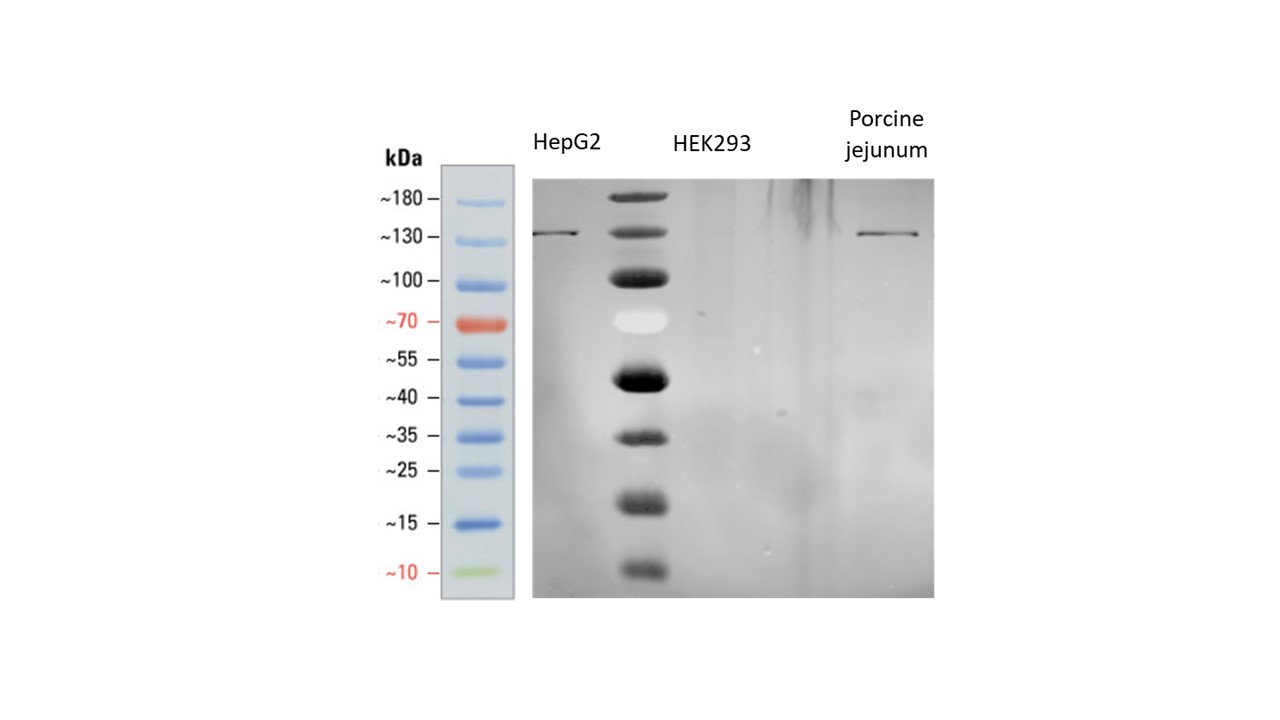

Supplement: Supplementary file 1 [file pharmaceutics-16-00116-s001.zip › Figure S3.jpg]

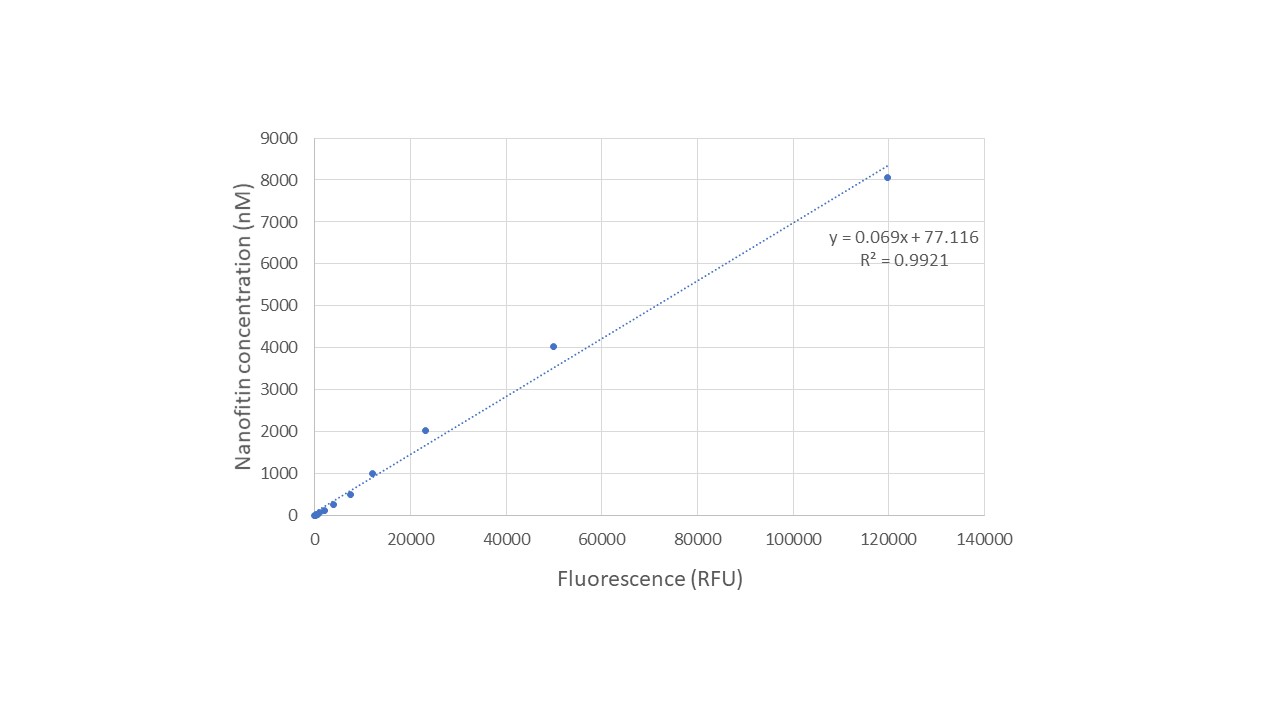

Supplement: Supplementary file 1 [file pharmaceutics-16-00116-s001.zip › Figure S4.jpg]

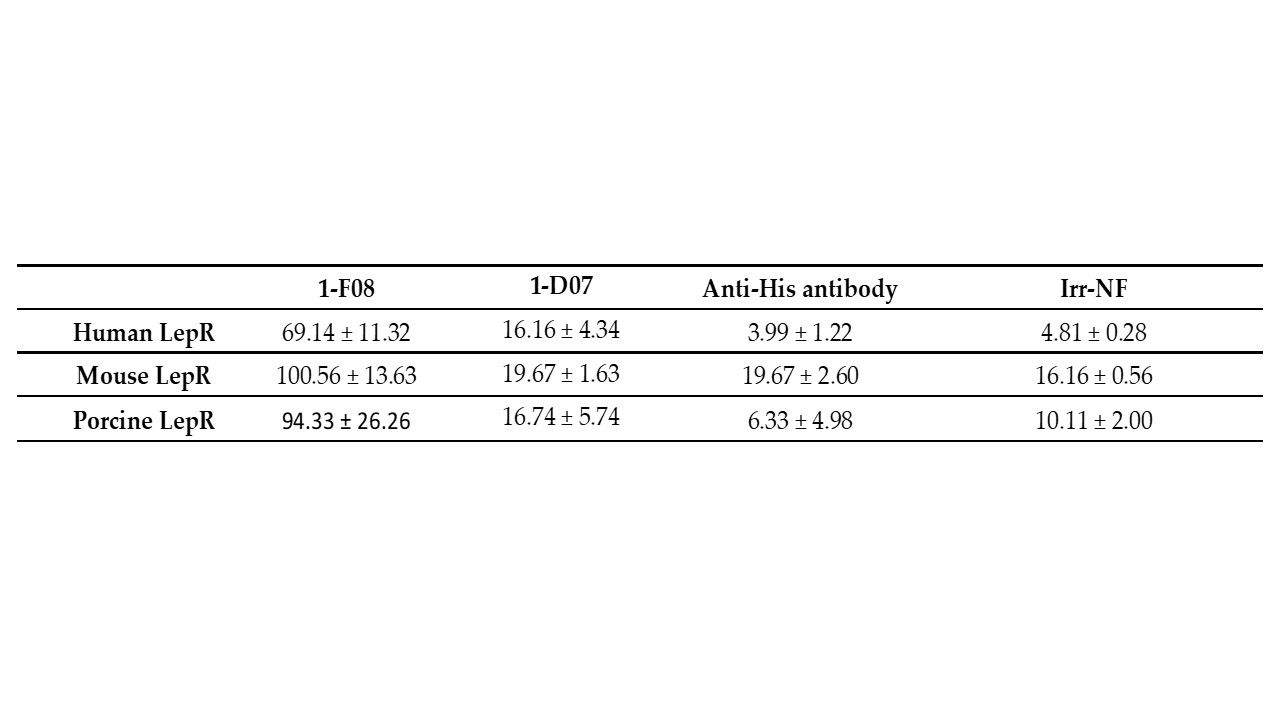

Supplement: Supplementary file 1 [file pharmaceutics-16-00116-s001.zip › Table S1.jpg]

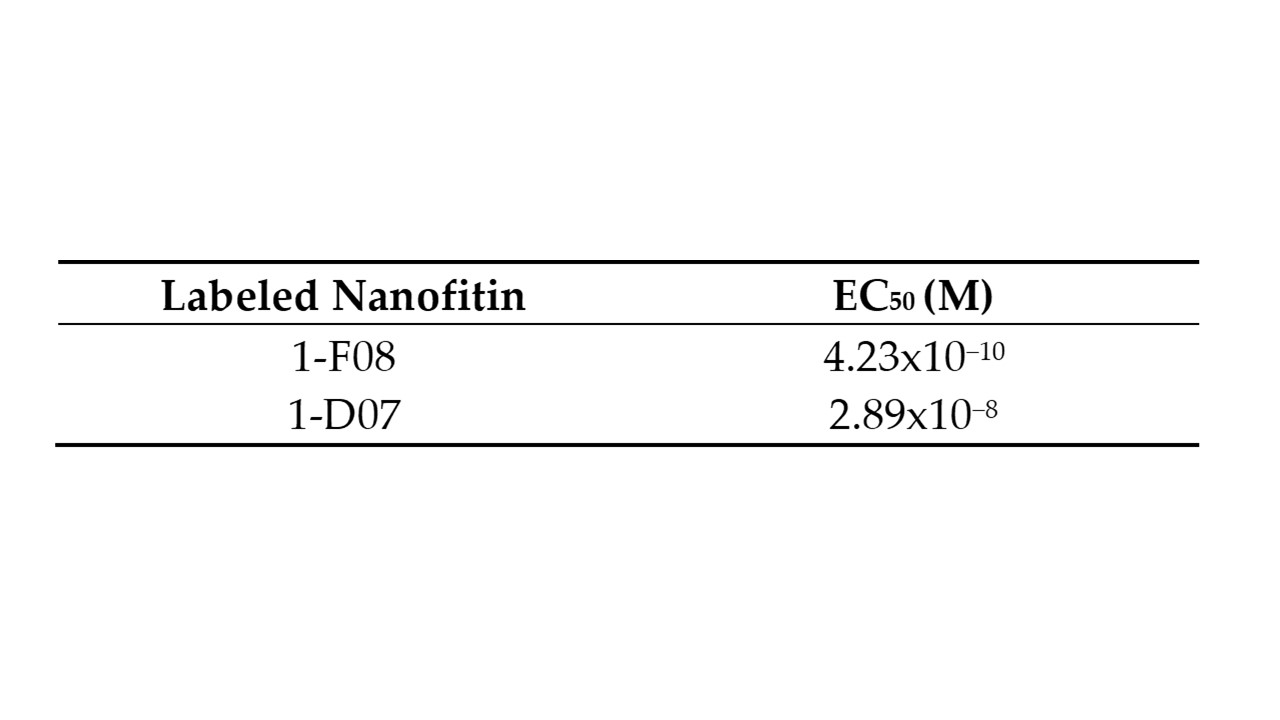

Supplement: Supplementary file 1 [file pharmaceutics-16-00116-s001.zip › Table S2.jpg]

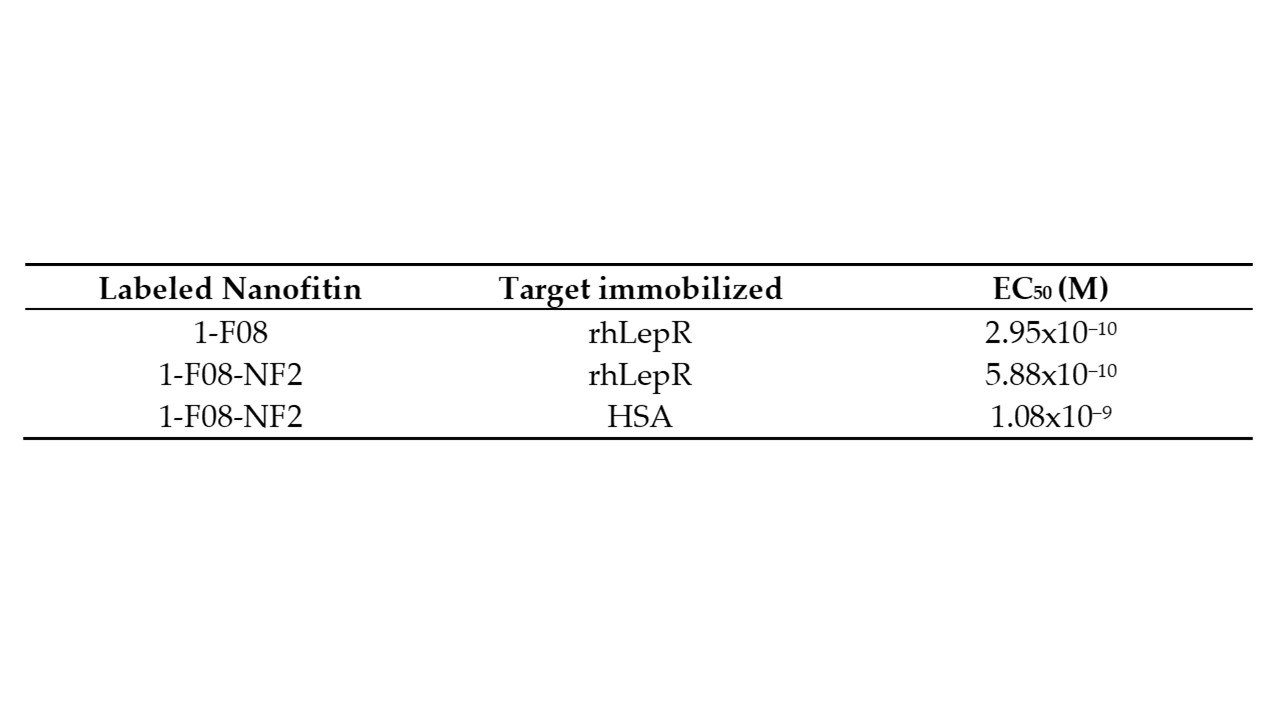

Supplement: Supplementary file 1 [file pharmaceutics-16-00116-s001.zip › Table S3.jpg]
